# Acute cellular rejection in pediatric heart transplant recipients is associated with abnormal left ventricular mechanics by CMR

**DOI:** 10.1186/1532-429X-18-S1-Q24

**Published:** 2016-01-27

**Authors:** Heynric Grotenhuis, Emile Nyns, Paul F Kantor, Anne Dipchand, Steven C Greenway, Shi-Joon Yoo, George Tomlinson, Rajiv Chaturvedi, Lars Grosse-Wortmann

**Affiliations:** 1 Pediatric Cardiology, The Hospital for Sick Children, Utrecht, Netherlands; 2 Pediatric Cardiology, The Wilhelmina Children's Hospital, Utrecht, Netherlands; 3 Pediatric Cardiology, Stollery Children's Hospital, Edmonton, AB Canada; 4 Department of Medicine, Toronto General Hospital and Mt. Sinai Hospital, Toronto, ON Canada

## Background

Acute cellular rejection (ACR) may lead to compromised graft function after pediatric heart transplantation (HTX). The purpose of this study was to describe systolic ventricular function in pediatric HTX and its relationship with ACR.

## Methods

18 combined cardiac magnetic resonance (CMR)/endomyocardial biopsies were performed in 14 HTX patients (11 male, age 13.9 ± 4.7 years (2.4-17.9 years); 1.2 ± 1.3 years (12 days-5.0 years) after HTX). Biventricular function and left ventricular (LV) circumferential strain, rotation and torsion by CMR were compared to 11 controls. To adjust for repeated measurements in 2 HTX patients, Wald-testing and Bayesian modeling were used for statistical analysis.

## Results

HTX patients showed reduced global LV circumferential strain (-13.5 ± 2.3%vs.-19.1 ± 1.1%, p < 0.01), basal strain (-13.7 ± 3.0%vs.-17.5 ± 2.4%, p < 0.01), mid-ventricular strain (-13.4 ± 2.7%vs.-19.3 ± 2.2%, p < 0.01), apical strain (-13.5 ± 2.8%vs.-19.9 ± 2.0%, p < 0.01), basal rotation (-2.0 ± 2.1°vs.-5.0 ± 2.0°, p < 0.01), apical rotation (8.6 ± 4.1°vs.8.3 ± 3.0°, p < 0.01) and LV torsion (6.1 ± 1.7°vs.7.8 ± 1.1°, p < 0.01) when compared to controls.

When comparing different degrees of ACR (Group A [grades 0-1 R]: 14 studies in 11 patients vs. Group B [grade 2R]: 4 studies in 3 patients), Bayesian modeling found high probabilities of 70-96% that RV ejection fraction, global strain, strain at all 3 LV levels and LV torsion were reduced in patients with grade 2 R rejection. Basal and apical rotation had highest sensitivity (91%;95%,CI:77%-100%) and specificity (100%;95%,CI:-100%) to discriminate between groups A and B.

## Conclusions

Pediatric HTX recipients, even in the absence of ACR, have decreased myocardial deformation by impaired LV circumferential strain, rotation and torsion. A further reduction is associated with ISHLT 2 R ACR. This pilot study suggests that myocardial deformation markers like basal and apical rotation may be non-invasive markers of significant ACR in pediatric HTX.

